# Experiencing the COVID-19 Pandemic at Age 85 and Over: An MIT AgeLab Study With the 85+ Lifestyle Leaders Panel

**DOI:** 10.1093/geroni/igab046.1068

**Published:** 2021-12-17

**Authors:** Taylor Patskanick, Lisa D'Ambrosio

## Abstract

The oldest of older adults remain at the highest risk of developing severe illness, requiring hospitalization, or dying if infected with COVID-19. As a result, the discourse about the COVID-19 pandemic has centered on short-term sacrifices to “protect” older adults. Yet much remains to be known about the prolonged impact of the pandemic on the over-85 age demographic. This symposium shares findings from a longitudinal, mixed methods study with the MIT AgeLab’s 85+ Lifestyle Leaders panel, a panel of octogenarians and nonagenarians convened since 2015. This symposium offers an update to a 2020 GSA session shared regarding the initial, cross-sectional work conducted in March 2020 with this panel. Findings will be drawn from a series of 85 interviews with 15 participants, 14 focus groups (x̅ participants=19.3), and three surveys (March 2020, N=28; August 2020, N=18; November 2020, N=16) conducted with the panel regarding the impact of the pandemic on this group over the past year. The first presentation covers the Lifestyle Leaders’ adoption and use of technology throughout the pandemic, with a focus on telehealth. The second takes an in-depth look at the unique lived experiences of Lifestyle Leaders living in senior housing communities during the pandemic. The third explores the Lifestyle Leaders’ perspectives on cultivating resilience and caring for their mental health while in a pandemic. Finally, the fourth presentation shares the Lifestyle Leaders’ experiences with social isolation and loneliness during the pandemic, with a focus on how family relationships and engagement in intergenerational programming have changed.

